# Purification and characterization of a serine protease (CESP) from mature coconut endosperm

**DOI:** 10.1186/1756-0500-2-81

**Published:** 2009-05-09

**Authors:** Leelamma M Panicker, Rajamma Usha, Samir Roy, Chhabinath Mandal

**Affiliations:** 1 National Institutes of Health, Building 10, Room 8C 209, Bethesda, Maryland-20892, USA; 2 Manovikas Biomedical Research & Diagnostic Centre, 482 Madudah, Plot I-24, Sector-J, EM Bypass, Kolkata-700 107, India; 3 DOEACC Centre (Bioinformatics Division, Jadavpur University), 4 Raja SC Mullick Road Kolkata-700032, India

## Abstract

**Background:**

In plants, proteases execute an important role in the overall process of protein turnover during seed development, germination and senescence. The limited knowledge on the proteolytic machinery that operates during seed development in coconut (*Cocos nucifera *L.) prompted us to search for proteases in the coconut endosperm.

**Findings:**

We have identified and purified a coconut endosperm protease (CESP) to apparent homogeneity. CESP is a single polypeptide enzyme of approximate molecular mass of 68 kDa and possesses pH optimum of 8.5 for the hydrolysis of BAPNA. Studies relating to substrate specificity and pattern of inhibition by various protease inhibitors indicated that CESP is a serine protease with cleavage specificity to peptide bonds after arginine. Purified CESP was often autolysed to two polypeptides of 41.6 kDa (CESP1) and 26.7 kDa (CESP2) and is confirmed by immunochemistry. We have shown the expression of CESP in all varieties of coconut and in all stages of coconut endosperm development with maximum amount in fully matured coconut.

**Conclusion:**

Since the involvement of proteases in the processing of pre-proteins and maintenance of intracellular protein levels in seeds are well known, we suspect this CESP might play an important role in the coconut endosperm development. However this need to be confirmed using further studies.

## Background

Proteases, being major regulatory enzymes, play a prominent house-keeping role in the cell physiology of all living systems. In plants, these enzymes execute an important role in the overall process of protein turnover in all stages of its life [1-6]. Seed development is an intricate process by which the seeds synthesize and store extensively a number of proteins, carbohydrates and lipids for subsequent use during seedling growth. Different types of proteases, which are active during seed development, are very scarcely studied, even though their importance in proteolytic processing of pre-proteins and regulation of intracellular protein levels are very well identified [7].

Coconut (*Cocos nucifera *L.), which belongs to the family of Palmae, is widely distributed in tropical countries. The properties of protein extracted from desiccated coconut showed that its protein content is comparable to soy protein in terms of composition and amount [8]. However, the research pertaining to the biochemical aspects of endosperm development are still lacking except few studies, which shows the chemical constituents of coconut endosperm and biochemical changes happening during the germination of coconut seeds suggests a metabolically active stage during seed development [9-14]. While investigating the presence of proteases in the developing endosperm, we have identified three types of these enzymes [15]. In the present paper, we report the purification and characterization of a serine protease from mature coconut endosperm (CESP), which possessed the BAPNA hydrolyzing activity. We also detected CESP in coconut endosperm during seed development and in different varieties of coconut using immunochemistry.

## Methods

For the purification of CESP, eighty grams of fully matured coconut kernel was used and the entire purification procedure was carried out at 4°C and the total enzyme activity and protein concentration at each level of purification was estimated.

### (i) Preparation of crude extract

Kernel was cut into small pieces, frozen in liquid nitrogen, powdered, homogenized in four volumes of ice-cold 20 mM TBS at pH 7.8 containing 150 mM NaCl (buffer A) using a wet grinder, strained through muslin cloth, centrifuged at 14,400 × *g *for 25 minutes, removed the upper creamy layer of fat and the clear crude extract was obtained by passing through glass wool.

### (ii) Ammonium sulfate fractionation and gel filtration

The crude extract was subjected to ammonium sulfate fractionation (30–60%) and the pellet was dissolved in a minimum volume of ice-cold buffer A and then loaded on to a Sephadex G-200 gel filtration column (105 cm × 2.3 cm) equilibrated with the buffer A. The fractions (25 to 35) with high specific activity were pooled.

### (iii) Phenyl-Sepharose Chromatography

Solid ammonium sulfate was added to the pooled fractions from the above step to yield a final concentration of 0.4 M (NH_4_)_2_SO_4 _and was then subjected to phenyl-Sepharose column (5 cm × 1.6 cm) pre-equilibrated with buffer A containing 0.4 M (NH_4_)_2_SO_4_. The bound enzyme was eluted using a gradient of buffer A containing 0.4 M to 0.0 M (NH_4_)_2_SO_4_. Fractions of 1 ml with high protease activity were pooled and dialyzed against buffer A without 150 mM NaCl (buffer B).

### (iv) DEAE-cellulose Chromatography

The pooled fractions were passed through DEAE-cellulose column (4.5 cm × 1.5 cm) equilibrated with buffer B. The enzyme was eluted using a gradient of 0 – 0.5 M NaCl in buffer B and the fractions with high protease activity were concentrated by dialyzing against buffer B using Centricon 10.

### (v) Arginine-Sepharose Chromatography

The dialysate from the previous step was loaded on this column (7 cm × 0.9 cm) equilibrated with buffer B and the bound enzyme was eluted using a gradient of (0–0.3) M NaCl in the same buffer.

### (vi) Biogel P60 gel filtration chromatography

Selected fractions from the previous step was pooled and concentrated to a volume of 0.5 ml and subjected to Biogel P60 column (50 cm × 1 cm), equilibrated with buffer A.

#### Enzyme activity assay

Protease activity was assayed during the purification process using a synthetic peptide N∞-Benzoyl DL-arginine p-nitroanilide (BAPNA) as the substrate. The reaction mixture contained 100 mM Tris HCl (pH 8.5), enzyme solution and 0.5 mM substrate in a total volume of 500 μl. Reaction was carried out at 37°C for one hour and stopped by an addition of 500 μl of ethanol. The liberated colored *p*-nitroaniline was measured at 405 nm using spectrophotometer (Hitachi U 2000). One unit of enzyme activity (EU) was defined as 1 μmole of *p*-nitroaniline liberated per hour under the conditions of assay. The enzyme activity was calculated using a *p*-nitroaniline molar extinction coefficient of 10,500 M^-1^cm^-1 ^at 405 nm.

#### Preparation of Antibody

Protein bands corresponding to the CESP proteolytic products of 26.7 kDa (CESP2) and 41.6 kDa (CESP1) were cut from the gel after SDS-PAGE and eluted using electro-eluter from Bio-Rad. Two rabbits were immunized independently (rabbit 1–26.7 kDa, rabbit 2–41.6 kDa) by giving three subcutaneous injections, for a total of 700 μg proteins into respective rabbits. After 7 days of the booster dose, rabbits were bled through the ear vein and tested for the presence and specificity of antibody by immuno-diffusion [16] and immuno-blot analysis [17]. Total IgG from the immune sera directed against 26.7 kDa polypeptide was isolated by 33% ammonium sulphate fractionation and DEAE cellulose ion-exchange chromatography.

#### Gel Electrophoresis

SDS-PAGE was carried out as described by Laemmli [18] on 12% polyacrylamide gel and non-denaturing PAGE on 7% polyacrylamide gel. Western blot analysis was done as described by Towbin [17] using immunized serum 1: 200. The detection of proteolytic activity on 12% polyacrylamide gel containing SDS and 0.25% gelatin was carried out as described by Heussen and Dowdle [19] except that Triton X-100 (1%) in Tris buffer pH 8.5 used for re-naturation of the gels. Gels were then incubated in the same buffer with out Triton X-100 for over night at room temperature.

#### Other methods

Absorbance at 280 nm was used for monitoring the protein during chromatographic elution. Total proteins in the pooled fractions were estimated using the Coomassie dye-binding assay [20]. Arginine-Sepharose and IgG-Sepharose affinity matrices prepared using cyanogen bromide method [21].

For the detection of CESP in different varieties (endosperms stored at -80°C about an year) and different developmental stages of coconut (fresh), crude extract was prepared from the endosperm of the coconuts collected from the identified palms.

## Results and Discussion

### Purification of CESP from the coconut endosperm

**Figure 1:**
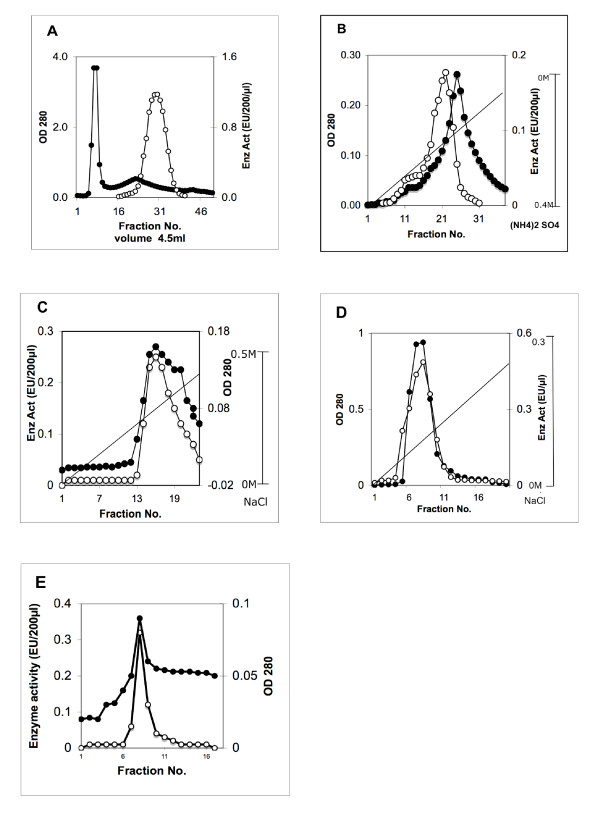
**Purification of the protease (CESP) from coconut endosperm**. The crude extract after ammonium sulfate fractionation was subjected to **(A)**, Gel filtration chromatography; **(B)**, Phenyl sepharose affinity chromatography; **(C)**, DEAE-cellulose chromatography; **(D)**, Arginine-Sepharose affinity chromatography; (**E**), Biogel P60 gel filtration chromatography. "Black circles" represents the absorbance of the protein at 280 nm and "white circles" represents the protease activity in Enzyme Units (EU). The straight line in figures B, C and D represents the gradient of salt concentration used for eluting the protease.

Purification steps of CESP are presented in Fig. 1 (A–D) and the summary of the whole process is tabulated in Table 1. The protease was purified approximately 41 fold by ammonium sulphate fractionation and gel filtration (Table 1). Later steps yielded only marginal increase in the fold purification leading to an overall recovery of 0.183 mg of protein with a specific activity of 14.42 EU/mg from 80 g of tissue. The enrichment of the CESP after the entire purification procedure resulted in 515-fold.

**Table 1:** Summary of Purification of CESP from coconut kernel.

**Steps**	**Total Protein (mg)**	**Total Activity (EU)**	**Specific Activity (EU/mg)**	**Yield (%)**	**Fold Purification**
1. Crude extract	1380	38.17	0.028	100	1

2. Sephadex G 200	16.51	19.09	1.16	50.02	41.42

3. Phenyl-Sepharose	1.36	5.95	4.37	15.57	156.1

4. DEAE-Cellulose	0.986	4.86	4.93	12.74	176.1

5. Arginine-Sepharose	0.260	3.21	12.37	8.43	441.8

6. Biogel P60	0.183	2.59	14.42	6.78	515

**Figure 2:**
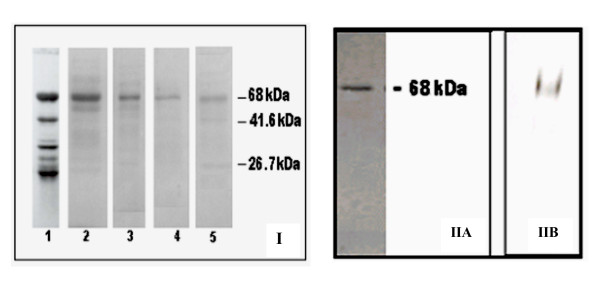
**Analysis of the protein by SDS/PAGE during enzyme purification and testing of the homogeneity of the purified CESP**. **2(I): **Proteins from different stages of the purification were analyzed by 12% SDS-PAGE. Different lanes were loaded with pooled proteins from different steps. Lane 1, crude homogenate (8 μg); Lane 2, gel filtration (4 μg); Lane 3, Phenyl-Sepharose (2.5 μg); Lane 4, DEAE-cellulose 2 μg; Lane 5, Arginine Sepharose (2 μg). **2(II)**: Analysis of the purified protease from Biogel P60 was carried out both by **(A) **SDS/PAGE on 12% polyacrylamide gel (1.8 μg) and **(B) **nondenaturing PAGE on 7% polyacrylamide gel (2 μg).

The Coomassie blue stained polyacrylamide gel from various stages of purification (Fig. 2(I)) showed that by DEAE step almost all the contaminating proteins were removed. A protein at 68 kDa region was the most prominent one observed. Two additional minor bands observed at 41.6 and 26.7 kDa in the last step of purification may be a result of autolysis, which could be removed again by gel filtration on Biogel P60 (Fig. 1E). In the absence of other proteins and endogenous substrates, the purified CESP might have undergone self-degradation. This was observed previously in other plant serine proteases [22-25].

### Homogenity and molecular size of the purified CESP

The SDS-PAGE analysis of the purified CESP immediately after Biogel P60 gel filtration step showed a single band at 68 kDa region (Fig. 2(II) A) and under non-denaturing conditions, gave a single broad protein band as shown in Fig. 2(II) B.

The molecular weight 68 kDa as determined by SDS-PAGE analysis (Fig. 2(II) B) and 69.5 kDa by gel filtration on Sephadex G-100 (see Additional file 1, Fig S1) indicate that CESP is a single polypeptide protease. The molecular weights of the serine proteases of cucumisin family and the latex peptidases also have been reported to be single polypeptide enzymes with molecular weight ranging between 60 and 80 kDa [25-28]. Molecular weights of the minor protein bands were estimated as 41.6 kDa and 26.7 kDa using SDS-PAGE analysis, suggesting that these might have arrived from the 68 kDa CESP.

### Substrate specificity and pH dependence of the purified CESP

**Figure 3:**
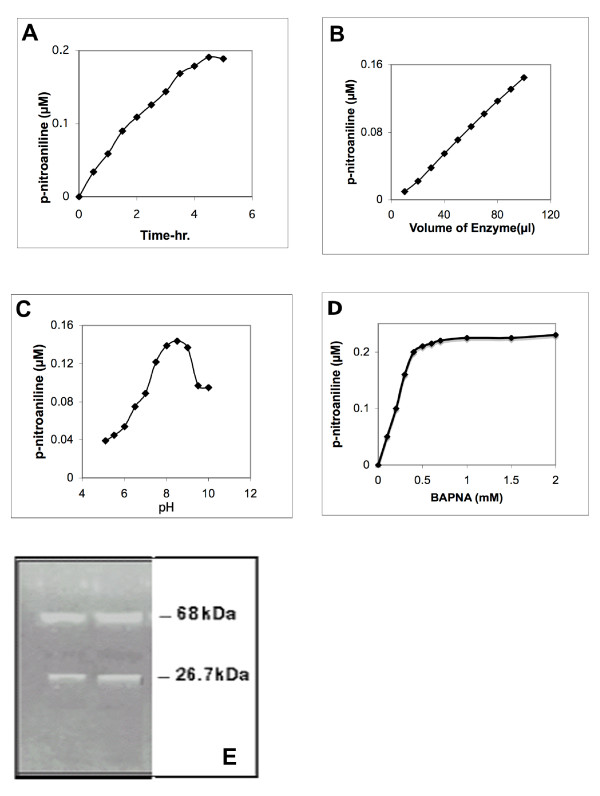
**Profile of BAPNA hydrolysis by the purified enzyme**. **(A) **50 μl of purified enzyme was assayed at pH 8.5 for BAPNA hydrolysis for different time periods up to 5 hrs using 50 mM BAPNA substrate **(B) **BAPNA hydrolysis was carried out for 1 hour with increasing volume of purified enzyme. **(C) **pH optimum of BAPNA hydrolysis by the purified CESP was studied in 100 mM buffers of varying pH. The buffers used were: Na Ac (5.1), MES (5.5, 6.0), PIPES (6.5, 7), Tris HCl (7.5 to 9) and CAPS (9.7, 10). (**D**) Measurement of Michaelis-Menten constant (Km): *p*-nitroaniline liberated was measured by varying substrate concentrations. Conditions of reactions are same as mentioned earlier. **(E) **SDS/PAGE zymogram analysis of the purified CESP, 2 μg (1^st ^lane) & 3 μg (2^nd ^lane) of the purified CESP after storage for one week.

The BAPNA hydrolysis was linear with time and increasing concentration of the enzyme (Figs. 3A, B). Similar to most of the serine proteases [26-29] the optimum enzyme activity using BAPNA was found to be at pH 8.5, above and below that pH the activity decreased (Fig. 3C). Km value of the enzyme was calculated as 0.2 mM for BAPNA (Fig. 3D), which is close to other serine proteases. Among the seven substrates tested, CESP hydrolyzed rapidly those that have arginine residue at the C-terminal side of the peptide bond (Table 2). This indicates that enzyme may be a trypsin-like protease. It also slowly hydrolysed Suc-Ala-Ala-Phe-MCA implying that CESP also has some chymotrypsin-like activity. However, the enzyme did not hydrolyse another substrate for chymotrypsin, Suc-Leu-Leu-Val-Tyr-MCA. The substrate specificity must be studied further to verify these conclusions, but similar specificity has been observed for some serine proteases like trypsin and other plant proteases like cuminisin and Q-SP [27,28].

**Table 2:** Hydrolysis of fluorogenic peptide substrate by the purified CESP

**Fluorogenic Substrates**	**Concentration** **used (mM)**	**Relative Fluorescence** **Intensity**
Boc-Leu-Arg-Arg-MCA	0.05	698.00

Boc-Val-Pro-Arg-MCA	0.05	583.50

Suc-Ala-Ala-Phe-MCA	0.05	120.70

Suc-Leu-Leu-Val-Tyr-MCA	0.05	0.060

Boc-Phe-Ser-Arg-MCA	0.05	625.85

Bz-Phe-Val-Arg-βNA	0.05	422.90

Bz-Leu-Leu-Glu-βNA	0.05	0.020

100 μl of reaction mixture containing 10 μl of purified enzyme, 0.05 mM of the substrates and 100 mM Tris HCl pH 8.5 was incubated at 37°C for 30 min and the reaction was stopped by the addition of 1.9 ml of ethanol. The product of hydrolysis was measured spectroflourimetrically (for AMC-Exc-380 nm, Em-460 nm and for βNA-Exc-335 nm, Em-410 nm).

### The effect of protease inhibitors, metal ions and complexes on CESP

Trypsin protease inhibitors TLCK and antipain, and chymotrypsin inhibitor TPCK strongly inhibited hydrolysis of BAPNA (Table 3). Other serine protease inhibitors Benzamidine and PMSF inhibited 50 and 43% respectively. However trypsin inhibitors, Aprotinin and SBTI did not affect the enzyme activity. Inhibition of CESP by a chymotrypsin inhibitor TPCK supports the observation on the substrate specificity that the protease may have some chymotrypsin-like activity. Pepstatin, metal ions, chelators and sulfhydryl-blocking agents did not inhibit the enzyme (Table 3). These results indicate that CESP is a serine protease [27].

**Table 3:** Effect of protease inhibitors, metal ions, chelators and sulfhydryl reagents on the activity of the purified CESP

**Inhibitor**	**Conc. (mM)**	**Enzyme** **Activity (%)**	**Ions/Complex**	**Enzyme** **Activity (%)**
No addition	-	100	No addition	100

TLCK	0.541	0	Ca	106

TPCK	0.568	13	Mg	115

Antipain	0.073	11	Zn	102

Benzamidine	0.641	51	Cu	79

PMSF	1.000	57	DTT	109

SBTI	0.0025	97	β-ME	103

Pepstatin	0.0364	93	EDTA	100

Aprotinin	0.0077	100	EGTA	103

The enzyme activity of purified CESP was measured in presence of inhibitors using 0.5 mM BAPNA in a reaction mixture (500 μl) as described earlier. The 100% activity is represented by 0.015 EU.

### Zymogram analysis of purified CESP on gelatin polyacrylamide gel

Zymogram analysis of the purified protease showed gelatin hydrolysis at positions that correspond to 68 and 26.7 kDa protein bands (Fig 3E). As the zymogram of the of crude extract prepared from fresh coconut, showed only one band at 68 kDa [15], it is possible that 26.7 kDa band is an autolysed product of the protease that has retained protease activity. The cleavage of gelatin into small peptides indicates CESP to be an endopeptidase, which can cleave the protein substrate at different locations to generate small peptides [26-29].

### Characterization of the polyclonal antisera raised in rabbits against (41.6 kDa and 26.7 kDa) polypeptides

Polyclonal antisera were raised in rabbits against the 41.6 kDa (CESP1) and 26.7 kDa (CESP2) polypeptides. Specificity against peptides was verified. Western blot analysis of step purification using these antisera recognized 68 kDa protein (see Additional file 1, Fig. S2 and Fig S3), Analysis of the purified CESP after storage for one week, gave two distinct bands in each case, one represented the intact CESP and the other represented either 41.6 kDa or 26.7 kDa polypeptides corresponding to CESP1 and CESP2 depending on the antisera used (see Additional file 1, Fig. S4).

### Zymogram and western blot analysis of the purified CESP after PAGE on gelatin gels

**Figure 4:**
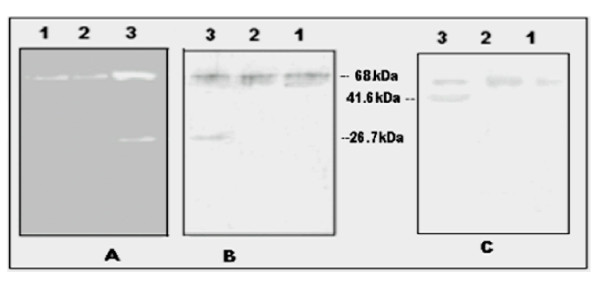
**Analysis of the CESP during purification by western blot with the antisera against the 26.7 kDa and 41.6 kDa polypeptide**. Comparison of the western blot analysis of the purified CESP with SDS/PAGE zymogram: SDS-PAGE was carried out on gelatin-containing polyacrylamide gel (12%) and the gel was cut into two pieces, one was used for zymogram analysis **(A)**. And the other part was electro-transfered to NC membrane. After transfer one part of the membrane was anlysed by western blot with antisera against 26.7 kDa CESP2 **(B) **and the other part with antisera against 41.6 kDa CESP1 polypeptide **(C)**. Lanes: 1, Crude extract 2, gel filtration fraction and 3, purified CESP.

CESP from various steps of purification were subjected to electrophoresis on gelatin gels and western blot analysis. Gelatin hydrolysis and western blots were compared simultaneously to find out the position of the bands as shown in Fig. 4. Zymogram showed two bands (at 68 kDa and 26.7 kDa) of gelatin-cleared zones (Fig. 4A, lane 3). These bands were at the same position as the two immuno-reactive bands observed on the western blot using the antisera against CESP2 (Fig. 4B, lane 3). The immuno-blot analysis using the antisera directed against 41.6 kDa polypeptide produced two bands that corresponded to intact CESP and CESP1 (Fig. 4C). But the 41.6 kDa region was not highlighted by zymogram analysis (Fig. 4A). In the case of CESP from the early Sephadex G 200 gel filtration step, the CESP1 and CESP2 could not be detected (Fig. 4, lanes 1 and 2). These results indicate that the CESP2 even after autolysis still retained the proteolytic activity but the bigger fragment did not exhibit proteolytic activity.

### CESP in different varieties of coconut (Cocos nucifera) and distribution during different stages of endosperm development

**Figure 5:**
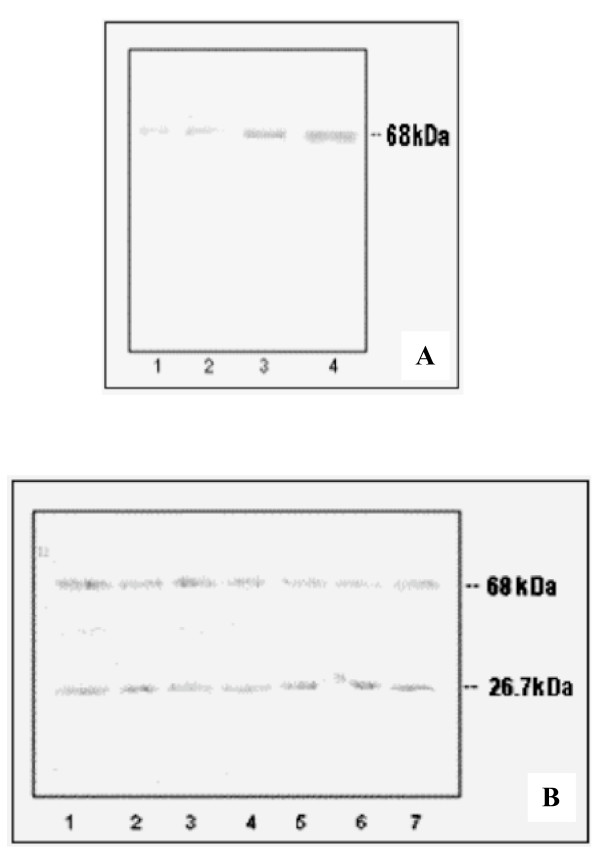
**Analysis of CESP for the expression of CESP during coconut maturation and varietal distribution using anti-CESP2**. **(A) **Endosperm crude extracts from the coconuts of different stages of development were subjected to western blot analysis. Lanes: 1, (6–7) months old coconut; 2, (8–9) months old; 3, (10–11) months old; 4, fully matured coconut or 12 months old coconut. 3 μg of protein loaded in each well. **(B) **Crude protein extracted from different coconut varieties were subjected to western blot analysis. Coconut varieties used are: Lanes 1, Hybrid-TXD; 2, Hybrid DXT; 3, Spikata; 4, Laccadive micro; 5, Chowghat dwarf orange (CDO); 6, Chowghat dwarf green (CDG); 7, West coast tall (WCT).

The western blot analysis showed that CESP2-antibody recognized the 68 kDa protein in the crude extract prepared from the developing endosperms (Fig. 5A). The increasing amount of CESP in the endosperm during the development from 6 months to maturation supports the result from the previous study [15]. The 68 kDa protein was also present in the crude extracts prepared from all coconut varieties (Fig. 5B). However 26.7 kDa peptide was also detected in these samples which indicate that the enzyme may be degraded during long-term storage of the kernel.

In general the involvement of proteases in the synthesis or degradation of proteins are clear. However, at this point in our studies, we do not know what is the function of CESP. There might be a possibility for a developmental specific function for CESP, as an endopeptidase more likely towards the processing of immature proteins and for the removal of regulatory proteins as and when it is needed during development.

## Conclusion

Identified enzyme (CESP) is a single polypeptide serine protease with approximate molecular mass of 68 kDa and possesses pH optimum of 8.5 for the hydrolysis of BAPNA. CESP is present in all varieties of coconut studied and in all stages of coconut endosperm development with maximum amount in fully matured coconut. But the physiological role of this alkaline protease is unknown. However, these finding may open up a lot of interest in the involvement of this protease in the area of development or in the storage and shelf life of coconut and its products.

## List of abbreviations used

**BAPNA**: N∞-Benzoyl DL-arginine p-nitroanilide; **TLCK**: N∞-p-Tosyl-L-Lysine chloromethyl ketone; **TPCK**: N∞-p-Tosyl-L-Phenylalanine chloromethyl ketone; **SBTI**: Soybean trypsin inhibitor.

## Competing interests

The authors declare that they have no competing interests.

## Authors' contributions

LMP designed the project, obtained funding support, performed all experiments, data analyzed, manuscript prepared, edited and done revisions. RU contributed to design experiments, data analysis and manuscript preparation. SR contributed to the antibody preparation. CM contributed designing the project, provided funding support, participated in the discussions and manuscript preparation. All authors have read and approved the final manuscript.

## Supplementary Material

Additional File 1 **Purification and characterization of a serine protease (CESP) from mature coconut endosperm**. Molecular weight measurement of CESP and characterization of the polyclonal antisera raised in rabbits against CESP1 and CESP2. Click here for file
