# Supplementary material for: Purification and characterization of a serine protease (CESP) from mature coconut endosperm
Source: BMC Res Notes. 2009 May 9;2:81. doi: 10.1186/1756-0500-2-81 (PMC2689245; doi:10.1186/1756-0500-2-81)
Supplement: Additional File 1 — Purification and characterization of a serine protease (CESP) from mature coconut endosperm. Molecular weight measurement of CESP and characterization of the polyclonal antisera raised in rabbits against CESP1 and CESP2. [file 1756-0500-2-81-S1.doc]

**Additional file 1**

Purification and characterization of a serine protease (CESP) from mature coconut endosperm

Leelamma M Panicker^1*^, Rajamma Usha^2^, Samir Roy and Chhabinath Mandal^3^

**^CONTENTS^:**

1. Isolation of CESP by immunoaffinity chromatography on

anti CESP2 IgG-Spharose

2. Legends to figures S1- S4

3. Figures S1-S4

***Isolation of CESP by immunoaffinity chromatography on anti CESP2 IgG-Sepharose***

The anti CESP2 IgG purified was coupled to sepharose and 6mg of IgG was immobilized per ml of Sepharose CL-4B. The partially purified protease after gel filtration on Sephadex G 200 was loaded on to IgG-Sepharose column (5 cm x 1.8 cm), equilibrated with 20mM Tris buffered saline pH 7.8. The column was washed thoroughly with the equilibration buffer to remove unbound proteins and the bound protease was eluted using 50 mM HCl. Fractions of 0.5 ml were collected into tubes, which contained 200 µl of 1 M Tris HCl pH 7.8. The fractions were assayed for protease activity using BAPNA as the substrate. The enzyme was eluted as a single peak and the pattern of elution is shown in Fig. S3A. All the fractions were having more or less same specific activity. SDS/PAGE analysis of the purified fraction showed a single band at 68 kDa region corresponding to CESP (Fig. S3B).

**Legends to Supplemental Figures**

*Fig. S1: Determination of Molecular weight of the purified protease (CESP) by Gel Filtration method.*

The molecular weight of CESP was measured by *Gel Filtration method using* Sephadex G-100 column and molecular weight standards used were 1. Aldose (158 kda), 2. BSA (67 kDa), 3. Eggalbumin (45 kDa) and 4. Chymotrypsinogen (25 kDa). Plotted log_10_ mol.wt against Ve/Vo.

*Fig. S2: Analysis of the CESP during purification by western blot with the antisera against the 26.7 kDa and 41.6 kDa polypeptide.*

SDS-PAGE was carried out with the enzyme fractions from different stages of purification and the proteins from the gel were transferred to NC membrane: Membrane was cut into two halves and treated each piece with antisera against either the CESP2 **(Ab1)** or CESP1 **(Ab2)** polypeptide**,** followed by immuno-detection. Lanes were loaded with fractions from different steps. Lanes: 1, Crude extract; 2, Gel filtration eluate; 3, Phenyl-Sepharose eluate; 4, DEAE-cellulose eluate and 5, purified CESP from arginine-Sepharose.

*Fig. S3: Analysis of CESP purified using immunoaffinity- chromatography.*

**(A)** Different fractions from the immunoaffinity column chromatography were assayed for protease activity using BAPNA as the substrate. **(B)** SDS-PAGE Lanes: 1, Crude extract; 2, Gelfiltration pooled; 3 & 4, Fractions from IgG-Sepharose column.

*Fig. S4****:*** *Western blot analysis of the purified CESP on storage:*

Purified protein after storage for one week at 4 ^0^ C was subjected to SDS/PAGE and western blot analysis. The antisera raised in rabbits against 26.7 kDa CESP2 protein **(Ab1)** and 41.6 kDa CESP1 protein **(Ab2)** were used separately for immuno-blot analysis.

**Fig. S1**

**Fig. S2**

**Fig. S3**

**Fig. S4**
